# Acquisition of new infection and clearance of type-specific human papillomavirus infections in female students in Busan, South Korea: a follow-up study

**DOI:** 10.1186/1471-2334-8-13

**Published:** 2008-01-30

**Authors:** Jin-Kyoung Oh, Young-Hee Ju, Silvia Franceschi, Wim Quint, Hai-Rim Shin

**Affiliations:** 1 Research Institute for National Cancer Control & Evaluation, National Cancer Center, 809 Madu, Ilsan, Goyang, Republic of Korea; 2 School of Public Health, Seoul National University, 28 Yeongeon, Jongro, Seoul, Republic of Korea; 3 International Agency for Research on Cancer, 150 Cours Albert-Thomas, Lyon, France; 4 DDL, Diagnostic Laboratory, Fonteynenburghlaan 7, 2275 CX Voorburg, the Netherlands

## Abstract

**Background:**

Little is known about the natural history of human papillomavirus (HPV) infection in Asian women.

**Methods:**

A follow-up study was conducted, with exfoliated cervicovaginal cells self-collected from, and questionnaires administered to 197 female students, aged 17–26 years, who had been already examined one and half years before. The presence of 25 HPV types was evaluated by a polymerase chain reaction-based assay.

**Results:**

The acquisition of new infection for any HPV type among 171 female students at risk who were negative at baseline, 60% of whom had remained as virgins, was 17.5% (95% confidence interval [CI]: 11.8–23.2). Among individual types, HPV16, 18 and 35 showed the highest rate of new infection. Women who had had first sexual intercourse (OR = 2.9; 95% CI: 1.0–8.8), or had changed sexual partners (OR = 2.9; 95% CI: 0.9–9.3) during the follow-up period showed a higher risk of new HPV infection than women who had remained virgins since baseline. The rate of new infection also tended to be higher in those who started, or continued smoking during the follow-up period, than in nonsmokers. Clearance of HPV infections since baseline examination was 80.6% (95% CI: 67.6–93.5), and did not differ between high-risk and low-risk HPV types or between single- and multiple-type infections.

**Conclusion:**

This study shows that the acquisition of new HPV infection among young women in the Republic of Korea is high and also the clearance is frequent. Self-collection of cervicovaginal cells is applicable to follow-up studies that include virgins.

## Background

More than half of sexually active women worldwide are infected by one or more genital human papillomavirus (HPV) types at some point in time [1], and the three-year cumulative incidence of HPV infection among young women in some Western countries is over 40% [2-4]. Although HPV infection is very common, 50% to 77% of HPV-positive women in cohort studies have been shown to clear the infection spontaneously in one year's time [1,2,5-8]. No studies, however, have investigated type-specific HPV incidence and clearance in Asian women. Hence, we followed up a group of female students in Busan, Republic of Korea, who had been evaluated for HPV prevalence one and a half years earlier [9].

## Methods

### Study design and participants

In 2002, The Korean National Cancer Center conducted a survey of HPV prevalence among female students in Busan. The study design has been described in detail previously [9]. Briefly, 672 female students from three different educational settings between 16 and 25 years of age participated, and provided self-collected samples of cervicovaginal cells in the first HPV prevalence survey in 2002. The HPV results would not be given to each participant due to the inability to provide a therapeutic agent in the case of positive results. About one and half years later, we mailed and/or made telephone calls to these same students, inviting them for a second examination.

To those who agreed, we mailed a package that contained an informed consent form, a self-administered questionnaire, instructions for self-collection of cervicovaginal cells in their home, and a self-collection kit (two Dacron swabs and a tube containing buffered methanol). The questionnaire requested information on changes in smoking and sexual habits since baseline examination. After three weeks, we made reminder calls to those who had not responded, and repeated the call once a week for three weeks, or until a response was obtained. We failed to contact 291 (43%) students due to changes in address or telephone number. Among the 381 students contacted, 19 (3%) refused to participate in the follow-up study, and 165 (25%) did not return the questionnaire and specimen. A total of 197 (29%) students sent back the questionnaire and cell sample. The median time interval between baseline and follow-up was 18.1 months (range: 17.1–19.7 months). All participants signed informed consent forms according to the recommendation of the Korean National Cancer Center Ethical Review Committee, which approved the study.

### Specimen collection

The students collected the cell samples themselves in the same way they had done at baseline [9]. They were asked to bend their legs and to introduce and gently rotate a Dacron swab (two swabs at follow-up, Medical Packing, CA, USA) in the vagina until reaching the cervix and to then place the swabs in a tube that contained 20 mL of buffered methanol (PreservCyt Soultion, Cytyc, MA, USA) without contaminating them. The specimens were stored at room temperature and delivered to the laboratory, where they were centrifuged at 3,000 × *g*, and the resulting pellets stored at -70°C until needed for HPV DNA testing.

### HPV testing

The method used for HPV testing was identical to that used at baseline [9]. Briefly, HPV DNA was isolated from the cytology specimen (MagNaPure Total Nucleic Acid system, Roche Diagnostics, Almere, Netherlands) and was amplified by use of a short PCR fragment (SPF)_10 _primer set. Amplification products first were tested by probe hybridization in a microtiter-plate assay (DEIA version 1) for the presence of HPV DNA. SPF_10 _amplimers from HPV-positive samples were subsequently analyzed by reverse hybridization in an HPV line-probe assay (LiPA Kit HPV INNO LiPA HPV genotyping assay, SPF-10 system version 1, Innogenetics, Gent, Belgium, manufactured by Labo Bio-medical Products, Rijswijk, Netherlands). PCR products hybridized, at high stringency, to these probes, generating a type-specific hybridization pattern. The HPV LiPA permits specific detection of 25 HPV types: HPV 6, 11, 16, 18, 31, 33–35, 39, 40, 42–45, 51–54, 56, 58, 59, 66, 68/73, 70, and 74. HPV 16, 18, 31, 33, 35, 39, 45, 51, 52, 56, 58, 59, 66, and 68/73 were considered high-risk types, and all the other were considered low-risk types. Samples that were SPF_10_-positive by DEIA but could not be identified by the type-specific oligonucleotide probes (LiPA) were classified as *HPV X *(undetermined HPV types) and categorized as low-risk types.

### Statistical analysis

The Pearson chi-square test and student T-test were used to compare data from participants and non-participants to the follow-up with respect to social and behavioral characteristics. We estimated the proportion of acquisition of new infection with specific HPV type(s) by calculation on the basis of the number of cases in which a given type was detected in woman whom that the corresponding type had not been detected at baseline. Infections were also classified as high-risk or low-risk HPV type infections [10]. Women infected with multiple-type infections that included one or more high-risk type were classified as high-risk infections. We used unconditional logistic regression to calculate odds ratios (ORs) and corresponding 95% confidence intervals (CIs) of new HPV infections by changes of sexual behavior and cigarette smoking. All analyses were conducted using STATA (version 9.0; StataCorp).

## Results

Table 1 shows the comparison of social and behavioral characteristics between participants and non-participants to the follow-up. There were no significant differences in mean age, smoking habits, alcohol drinking, sexual intercourse and HPV-positivity between two groups.

**Table 1:** Comparison of selective characteristics at baseline between groups of participants and nonparticipants to the follow-up.

**Characteristics**	**Nonparticipants**	**Participants**	**P-value**
Age (mean)	18.4 Year	18.5 Year	0.366
Smoking	26%	18%	0.062
Alcohol drinking	47%	51%	0.867
Age at menarche (mean)	13.3 Year	13.2 Year	0.199
Sexual intercourse	26%	21%	0.066
Age at first sexual intercourse (mean)	18.3 Year	17.9 Year	0.326
Lifetime multiple sexual partners	13%	12%	0.295
HPV positive	16%	13%	0.381

Among 197 students who participated in the follow-up study, 26 (13.2%) were positive for HPV DNA at baseline. Table 2 shows the acquisition of new HPV infection by type(s) among students at risk who were HPV-negative for the corresponding type(s) at baseline. New infection with any type of HPV was observed in 30 women (17.5%) one and half years later. Acquisition of new infections was highest for HPV 16, X, 18 and 35.

**Table 2:** Acquisition of new human papillomavirus (HPV) infection during one and half years, in female students negative for the corresponding HPV type(s). Busan, Korea

**HPV type**	**No. of women at risk (HPV-negative at baseline)**	**No. of new Infections at follow-up**	**Acquisition of new infection, % (95% CI)**
High-risk			
16	195	17	8.7
18	196	8	4.1
31	195	3	1.5
33	196	1	0.5
35	197	6	3.0
39	196	2	1.0
45	197	0	0.0
51	190	1	0.5
52	195	4	2.1
56	196	0	0.0
58	196	2	1.0
59	196	2	1.0
66	196	2	1.0
68	194	2	1.0
*Overall HR*	181	23	*12.7 (7.9–17.6)*
Low-risk			
6	197	4	2.0
11	196	0	0.0
34	197	0	0.0
40	197	0	0.0
42	197	0	0.0
43	197	2	1.0
44	197	1	0.5
53	196	1	0.5
54	196	3	1.5
70	196	0	0.0
74	195	1	0.5
X	190	13	6.8
*Overall LR*	187	15	*8.0 (4.1–11.9)*
**Any type**	**171**	**30**	**17.5 (11.8–23.2)**

X: undetermined HPV type; CI: confidence interval; HR: high-risk; LR: low-risk.

Table 3 shows the new HPV infections by changes in sexual behavior or smoking habit. After adjustment for age, the incidence of infection was higher in students who had had first intercourse (OR = 2.9; 95% CI: 1.0–8.8) or had changed sexual partner (OR = 2.9; 95% CI: 0.9–9.3) during the follow-up period compared to those who had remained virgins since baseline, although the associations were borderline statistical significance. After adjustment for age and for changes in sexual habits, the incidence of infection was also higher in those who started (OR = 6.1; 95% CI: 0.9–40.8) smoking during the follow-up period than in nonsmokers.

**Table 3:** New infection of human papillomavirus (HPV) among 171 HPV-negative female students according to changes in sexual or smoking habits between baseline and follow-up. Busan, Korea

**Baseline**	**Follow-up**	**No. of women**	**New infection (%)**	**OR (95% CI)**
**Sexual intercourse**				
No	No	118	15 (12.7)	1.0
No	Yes	20	6 (30.0)	2.9 (1.0–8.8)
Yes	Yes, same partner	7	1 (14.3)	1.1 (0.1–10.3)
Yes	Yes, new partner	17	5 (29.4)	2.9 (0.9–9.3)
Not reported	Yes	9	3 (33.3)	3.4 (0.8–15.2)
Current smoking*				
No	No	131	16 (12.2)	1.0
No	Yes	6	3 (50.0)	6.1 (0.9–40.9)
Yes	Yes/No	12	5 (41.7)	3.3 (0.7–14.6)
Not reported		22	6 (27.3)	2.8 (0.9–8.4)

* ORs for changes in sexual habits were adjusted for the two age groups (years of 15–18 and 19–24); ORs for changes in smoking habits were adjusted for the two age groups and the five different categories of changes in sexual habits. OR: odds ratio; CI: confidence interval.

Table 4 shows clearance of HPV infections by type and multiplicity. Out of 36 HPV infections with 17 different HPV types or *HPV X *detected at baseline in 26 students, 80.6% were cleared at follow-up, one and half years later. Clearance was not statistically different between low- and high-risk HPV types, or single- and multiple-type infections, though the number of cases was limited. If the 26 HPV-positive women are used as a denominator, 26.9% (7/26) became HPV-negative, 50.0% (13/26) cleared their baseline infection(s) but acquired new HPV types, and 23.1% (6/26) remained positive for the same HPV type(s) as at baseline (including three students with persistent *HPV X *infection) (data not shown).

**Table 4:** Clearance of 36 human papillomavirus (HPV) infections during one and half years, among 26 female students HPV-positive at baseline. Busan, Korea

**Type(s)**	**No. of infection**	**Clearance (%)**
HPV 16	2	1 (50.0)
HPV 31	2	1 (50.0)
HPV 51	7	7 (100.0)
Any HR	23	20 (87.0)
LR only	13	9 (69.2)
Single type	19	15 (78.9)
Multiple type	17	14 (82.4)
Any HPV	36	29 (80.6) (95% CI: 67.6%–93.5%)

HR: high-risk; LR: low-risk; CI: confidence interval.

## Discussion

The prevalence of HPV infection in female students in Busan, Korea, at baseline was 15.2% [9]. In the follow-up study, 17.5% of 171 students at risk who were HPV-negative at baseline became positive. Reinfection with a new HPV type was also frequent (50% of 26 HPV-positive students). The acquisition of new HPV infections was approximately 30% in women who became sexually active or changed sexual partner during the follow-up period. Interestingly, 13% of the 118 students who reported that they never had penetrative sexual intercourse became HPV-positive at follow-up. Modes of HPV transmission, besides penetrative sexual intercourse, have been suggested [4,11], but, although the questionnaire was self-administered and full confidentiality was assured, we cannot rule out inaccuracies in the reporting of sexual behavior. In addition, there may be a possibility to be contaminated or false positive for HPV test of the samples with never having a penetrative sexual intercourse, even though it is very little or rare.

Acquisition of new HPV infection is common, particularly among sexually active young women, and the incidence appears to be higher for oncogenic than for non-oncogenic types [6,12]. A prospective study in Brazilian women showed that there were 1.3% new infections per month, with 38% cumulative positivity after one and a half years [5]. In Canadian university students, the incidence rate was 1.9% per month, and the cumulative rate was 18.0% at one year and 36.4% at two years of follow-up [12]. In another study of Canadian women, the overall infection incidence was 11.1% per year, with the highest rate (25.0%) in the 15–19-year age group [8]. The rate of new HPV infections in young women in the United States was 2.9% per month [6], and the three-year cumulative incidence was over 40% [2-4,11]. In our study, the rate of acquisition of new infections for any HPV type per 1000 woman-months was estimated as 9.8 (95% CI: 6.6–14.0) (data not shown). Considering the longer follow-up interval than other studies, this study suggests that HPV incidence in Korean female students is relatively lower than that in corresponding populations in Western countries.

Current knowledge on the relationship between smoking and incidence of HPV infection is limited, with previous studies reporting inconsistent results [11,13,14]. In our baseline study, smoking was one of the strongest risk factors for HPV-positivity in female students (ever-smokers vs. never-smokers, OR = 3.8; 95% CI: 1.9–7.5) [9]. In current follow-up study, smoking was again a predictor of HPV incidence, although, after adjustment for changes in sexual habits, the association was not statistically significant. Moreover, clearance of HPV infection was more frequent in women who never smoked than in women who ever smoked, but the association was not statistically significant (data not shown).

HPV infection is common, but it is highly transient. In our study, 80% of infections cleared and 77% of students who were HPV-positive at baseline became negative for the HPV type(s) present at baseline after one and a half years. Other studies also showed that the majority of HPV infections clear after one or two years [2,5-8,12,15]. In a population-based five-year follow-up study in Colombian women, 77% of HPV infections cleared after one year and 93% cleared after five years; clearance was most frequent in the first six months of follow-up [7]. Other cohort studies also show 50%–75% clearance of HPV infections after one year [2,5,6,8].

A self-collected Dacron swab sample of cervicovaginal cells is a technically feasible alternative to clinician-administered cervical cell collection in natural history studies of HPV and cervical cancer [16]. Our previous study, the baseline study, also showed that almost self-collected cervicovaginal samples were very adequate for test (β-globin positive in 99.1% of female students) [9].

Our current study has strengths and weaknesses. It is the first study to apply self-collection of cervico-vaginal cells and a very sensitive HPV testing method in a follow-up study that included many virgins, and to show high rate of acquisition of new HPV infection in young Asian women. The HPV testing method that we used at baseline and also at follow-up was highly sensitive but there could be undetected infections. New infections could be infections that were previously there and were missed, and cleared infections could be due to errors in the follow-up sample. However, these limitations are also important in this study as other studies.

Unfortunately, despite repeated efforts, traceability and compliance among female students was low. Only 57% (381/671) of female students were available to contact and 52% (197/381) of those who contacted, participated this study. Furthermore, there were only 36 baseline infections available for the analysis of clearance. Thus, there was limitation to detect significant associations between risk factors and HPV acquisition or clearance. However, when we compared the characteristics (i.e., age, HPV positivity, sexual, smoking and alcohol drinking habits at baseline) of study subjects who were and were not included in the follow-up study, they did not differ significantly (Table 1). Moreover, based on the Korean National Health and Nutrition Survey and other studies [17,18], the health behaviors (i.e., smoking and alcohol drinking habits and sexual intercourse) of study participants were similar to those of any other Korean girls of these ages. Therefore our present findings on HPV acquisition and clearance are not highly biased.

## Conclusion

In summary, we investigated acquisition and clearance of HPV infection with 197 followed-up female students in Busan, Korea. This study shows that the acquisition of new HPV infection among young women in the Republic of Korea was high and also the clearance was frequent. Self-collection of cervicovaginal cells is applicable to follow-up studies that include virgins.

## List of abbreviations

HPV, Human papillomavirus; OR, Odds ratio; CI, Confidence interval; PCR, Polymerase chain reaction

## Competing interests

The author(s) declare that they have no competing interests.

## Authors' contributions

JKO carried out the survey, analyzed and interpreted data, and drafted the manuscript. YHJ participated in the design of the study and carried out the survey. SF involved in designing the survey, drafting the manuscript and revising it critically for important intellectual content. WQ carried out the laboratorial works and drafted the manuscript. HRS conceived of the study, and participated in its design and coordination and helped to complete the manuscript. All authors read and approved the final manuscript.

## Pre-publication history

The pre-publication history for this paper can be accessed here:


